# Research-Driven Guidelines for Delivering Group Exercise Programs via Videoconferencing to Older Adults

**DOI:** 10.3390/ijerph19137562

**Published:** 2022-06-21

**Authors:** Tracy L. Mitzner, Elena T. Remillard, Kara T. Mumma

**Affiliations:** Center for Inclusive Design and Innovation, Georgia Institute of Technology, Atlanta, GA 30318, USA; elena.remillard@design.gatech.edu (E.T.R.); kara.mumma@design.gatech.edu (K.T.M.)

**Keywords:** telehealth, telewellness, older adults, mobility disability, exercise, social connectedness

## Abstract

Telehealth holds much potential for supporting older adults’ physical and social health. In particular, telewellness interventions to support the physical and social wellness of older adults are needed to overcome participation barriers with in-person programs. This paper presents guidelines for delivering telewellness interventions to older adults, which were informed by a human factors approach to developing a Tele Tai Chi intervention for older adults with mobility disabilities, including reviewing user needs literature and conducting user-centered needs assessment research. From these findings, we developed a protocol and support materials for delivering a telewellness intervention and conducted a feasibility study. We also established an adaptation committee to provide recommendations on the intervention. The outcome of our human factors approach was the establishment of research-driven design guidelines for delivering group exercise programs to older adults using videoconferencing. The guidelines provide direction for designing a telewellness protocol, supporting remote participation, and promoting socialization and engagement. These guidelines can be used to deliver interventions that increase access to socially-engaging, physical activity programs for older adults, which can ultimately help support their physical health, mental health, and quality of life.

## 1. Introduction

Telehealth has immense potential to promote and improve health outcomes for older adults (i.e., 65 years of age and older) with and without disabilities, through telemedicine and telewellness applications. Whereas telemedicine refers specifically to remote clinical services, ‘telewellness’ applications focus on health maintenance and chronic disease prevention. Physical activity and social interventions are two types of telewellness applications that can improve the health of older adults [1], a patient population growing in numbers. In 2019, the older adult population accounted for 16% of the United States population and is expected to grow to 21.6% in 2040 [2]. A significant number of older adults have a mobility disability, defined as “serious difficulty walking or climbing stairs.” Mobility disability is the most prevalent disability in the older adult population, impacting over 15% of older adults (ages 65–74), 26% of those ages 75–85, and 48% of those ages 85+ [3].

Many older adults, and in particular those with disabilities, are not receiving the benefits of physical exercise and social connectedness. Physical activity is a critical component to maintaining one’s health; it is ranked as a leading health indicator for individuals of all ages [4] and is often prescribed as a part of rehabilitation [5,6,7,8] and for managing and preventing health conditions [9]. Physical activity increases average life expectancy and reduces the risk of stroke, falls, and many chronic conditions [10,11,12]. Social activity, or social engagement and connectedness, has also been implicated in protecting both physical and mental health [13,14]. Social connectedness has been shown to lower anxiety [15], sustain cognitive and physical well-being, and reduce mortality risk [16,17,18], whereas social isolation and loneliness have been consistently linked to worse cardiovascular and mental health outcomes [19]. Older adults with adequate social connectedness have been shown to have a 50% greater likelihood of survival compared with those with poor social connectedness [17].

Most older adults do not meet the recommended guidelines for physical activity, and 28–34% of adults 65 to 74 years of age are inactive [12]. Likewise, almost one-quarter of community-dwelling older adults are considered socially isolated, and 43% of those 60 and older report feeling lonely [20]. Telehealth can provide a more accessible option for supporting older adults’ physical and social health. Research has documented much success for telehealth applications to support physical activity. They have been shown to be acceptable and effective for a variety of rehabilitation programs [21,22,23,24,25], including those for people with various degrees of mobility disability [26]. Telehealth physical activity interventions with a social component have been less studied, yet there are some promising findings regarding efficacy for decreasing feelings of loneliness and depression [27].

Physical activity interventions that also improve social connectedness may decrease loneliness and increase long-term physical activity participation [28]. Physical activity programs with a social component also motivate class participation and tend to have greater adherence [29,30]. Studies suggest that incorporating socialization could also help increase adherence and effectiveness of web-based exercise programs [31]. Moreover, older adults report that socialization is a major reason for participating in community-based programs [32]. Given potential compounded benefits from a telewellness intervention that includes both physical activity and social engagement, this represents an opportunity for a new direction to improve the health and quality of life for older adults on multiple dimensions.

However, there are challenges to delivering telehealth interventions to older adults, given the wide range of physical, cognitive, and sensory capabilities and limitations. Many older adults have mobility disabilities that need to be considered and accommodated in telehealth interventions. Age-related changes in cognitive and sensory abilities can impact telehealth accessibility as well [33]. For example, declines in visual acuity and the visual field can make it difficult for older adults to recognize fine details of icons or pointers used in interfaces [34,35]. Similarly, many older adults experience age-related hearing loss, which can impact their ability to effectively understand information and communicate. To increase accessibility, technologies need to be able to accommodate age-related sensory declines [33] and provide the training that is needed and wanted [33,34,36,37].

Ensuring safety is an additional challenge for older adults participating in remote exercise classes. Safety risks need to be thoroughly assessed and minimized since the instructor may not be able to see the participant’s entire body and cannot physically support them. Safety concerns, including the risk of falling, have been documented in remotely delivered assessments, such as Timed Up and Go, the 30-s chair stand test, and the 10-min walk test [23]. Similarly, a review of telerehab interventions for people with multiple sclerosis highlights that web-based exercise interventions often lack direct supervision of the participants’ movements and are limited in their ability to do so with the fixed camera [31].

Telehealth technology-related issues for this population primarily stem from older adults having lower levels of technology adoption and less technology experience than their younger counterparts. In 2016, only 67% of older adults (65+ years of age) reported use of the Internet, compared to 90% of the general adult population [38]. While technology and internet access remain a barrier for many [39], a growing number of older adults have learned how to use, and have become accustomed to, videoconferencing software, such as Zoom, to socialize with friends and family and participate in healthcare appointments remotely as a result of the COVID-19 pandemic. Data from an AARP (2021) survey showed that in 2019, about half of those surveyed had never used video chat, but by 2020, 70% had, with one in three using video chat weekly [40]. In addition to limited technology familiarity, other common technology issues with telehealth interventions include Wi-Fi availability as well as limited internet bandwidth, which can result in low-resolution images, audio delays, and the inability to connect [41,42]. To address some of these telehealth technology barriers, some studies have suggested solutions including implementing a training session in advance of the program to help participants get familiar with the technology and contacting participants by phone when tech issues arise.

To be inclusive of individuals with a wide variety of technology experiences, telehealth applications should be intuitive and supported by training and instructions. Considering the significant potential benefits of telewellness classes for older adults, there is a need for research-driven guidelines to support their delivery and overcome the potential challenges.

### Objectives

We used a human factors approach and methods to develop and test a telewellness protocol delivering an evidence-based exercise intervention (Tai Chi for Arthritis–seated version) via Zoom to older adults with mobility disabilities. This paper presents the approach we used to develop the Tele Tai Chi clinical trial, which also informed our development of guidelines for delivering group exercise classes via videoconferencing for older adults.

## 2. Methods

We developed a Tele Tai Chi intervention for older adults with mobility disabilities as a part of a clinical trial (TechSAge Tele Tai Chi for People Aging with Mobility Disabilities; ClinicalTrials.gov Identifier: NCT04696887). Our human factors approach included reviewing user needs literature and conducting user-centered needs assessment research.

The user needs literature review focused on understanding how social and physical activity have been defined in the literature and how these constructs have been targeted through evidence-based interventions, in particular those delivered via technology [1]. The review highlighted the implications for persons aging with mobility and sensory disabilities. Findings from the user needs literature review highlighted the need for home-based physical activity and social interventions for older adults, which can be modified appropriately for those with mobility disabilities. The findings also pointed to the need to increase access to evidence-based programs (EBPs), which are research-supported, packaged programs to promote health and wellness. EBPs are required to meet a number of standards, including published research studies that demonstrate benefits and safety, and the successful translation of the program into community organizations [43].

As a part of our needs assessment, we conducted a subject matter expert (SME) study with 12 exercise instructors who had experience working with adults aging with mobility disabilities to understand the factors that contribute to exercise class successes, class challenges, and methods for overcoming the challenges [44]. In addition, the exercise instructors gave insights into the requirements for doing an exercise program at home. Responses were transcribed verbatim and coded independently by three researchers using a thematic approach (Reliability = 88% Agreement). The findings pointed to several exercise class design recommendations for Telewellness classes, including creating a positive group dynamic that affords camaraderie among class members and providing opportunities for socialization commensurate to the socialization that occurs naturally before and after in-person exercise classes. The SMEs also discussed considerations to ensure the safety of participants while exercising remotely. They stressed the importance of offering a variety of exercise adaptations and modifications to accommodate varying levels of ability, which is especially critical when working with adults aging with mobility disabilities who may have diverse abilities and limitations. They also described how the exercise space or chair itself can pose an obstacle or fall hazard. The class content and safety recommendations of the SMEs were incorporated into the protocols and materials for the TechSAge Tele Tai Chi clinical trial.

We also engaged target users in our needs assessment. We conducted a study with 14 adults aging with a self-reported mobility disability (50–70 years of age) to assess their attitudes toward a range of technologies in the context of telehealth (i.e., supporting social engagement, healthcare provider access, and physical activity [45]). This was a mixed-methods study, in which we collected both quantitative (e.g., technology acceptance questionnaire) and qualitative data (i.e., structured interviews). The quantitative data (i.e., technology acceptance questionnaires [45]) showed that participants were generally open and positive about accepting technology for various types of telehealth (e.g., healthcare, physical activity). The interviews were transcribed verbatim and coded independently by three researchers using thematic analysis and a combined top-down and bottom-up approach (Reliability = 98% Agreement). The qualitative data revealed that participants perceived the technologies to be useful and have many benefits, including the feeling of “being there” by enabling the viewing of facial expressions and the environment of the other person. Nevertheless, participants had concerns about security and privacy, as well as concerns that the technologies could be difficult to use and difficult to learn to use. Based on these findings, we developed education and training recommendations for facilitating acceptance and adoption of telehealth technologies, including educating users about privacy features and videoconferencing etiquette and providing basic training for using the technology. These recommendations were also incorporated into the protocols and materials for the TechSAge Tele Tai Chi clinical trial.

Once we developed the first iteration of a protocol and materials for the TechSAge Tele Tai Chi study, we conducted a mixed-method feasibility study with adults aging with mobility disabilities [46]. The goal of the feasibility study was to assess user attitudes and implementation requirements of a Tai Chi class delivered via videoconferencing. Participants were 19 adults aging with a mobility disability (age *M* = 61.2 ± 10.81), who engaged in a seated Tai Chi exercise class via videoconferencing software. User attitudes were assessed with questionnaires and a semi-structured interview, whereas implementation requirements were assessed with a semi-structured interview and observational methods. Interviews were transcribed verbatim and coded independently by two researchers using thematic analysis and a combined top-down and bottom-up approach (Reliability = 84% Agreement). Participants perceived the Tele Tai Chi class to be useful, easy to use, and enjoyable. Participants also expressed positive attitudes about the telehealth Tai Chi class and its perceived physical, emotional, and social benefits. The implementation requirements that were identified included sufficient font size for all provided text, adjustable volume, exercises with tailored modifications, and conditions for environmental design. The protocol and materials were revised based on the findings from the feasibility study.

We then conducted usability testing to identify the optimal videoconferencing platform and settings for delivering the Tele Tai Chi protocol. We iteratively evaluated three videoconferencing platforms including: OneClick.chat, BlueJeans, and Zoom. Participants included nine older adults (age *M* = 68.5 ± 6.31), including three individuals with mobility disabilities. In the testing sessions, participants used the support materials we created to join and participate in a mock Tele Tai Chi class on one of the three platforms: OneClick.chat (*n* = 4), BlueJeans (*n* = 2), and Zoom (*n* = 3). Researchers observed and noted any user difficulties. After the class, we conducted a brief interview to assess ease of use and what is needed for additional support. Through usability testing, we identified the best platform and settings for the intervention (Zoom) and developed a list of key considerations to guide others in selecting and optimizing a videoconferencing platform for the delivery of telewellness classes. We also assessed the needs for orienting participants with remote participation and needs for technical support and assistance.

Finally, since we were translating an in-person intervention to videoconferencing, we established an adaptation committee to ensure relevant expertise for translating and adapting the Tai Chi intervention without impacting its fidelity. The adaptation committee contained target population representatives, a researcher with expertise in exercise and aging with disability (PhD, MPT, ATP), a researcher with expertise in designing technology for older adults (PhD), and a representative from the Tai Chi for Health Institute (Master Trainer). All committee members provided guidance on promoting socialization and engagement in the classes and provided feedback on the final protocol and support materials. The researcher with expertise in exercise and aging with disability (PhD, MPT, ATP) and the representative from the Tai Chi for Health Institute (Master Trainer) provided recommendations on class structure (e.g., instructor training, modifications) and safety (i.e., requirements for exercise readiness, consent/liability).

## 3. Results

**Figure 1:**
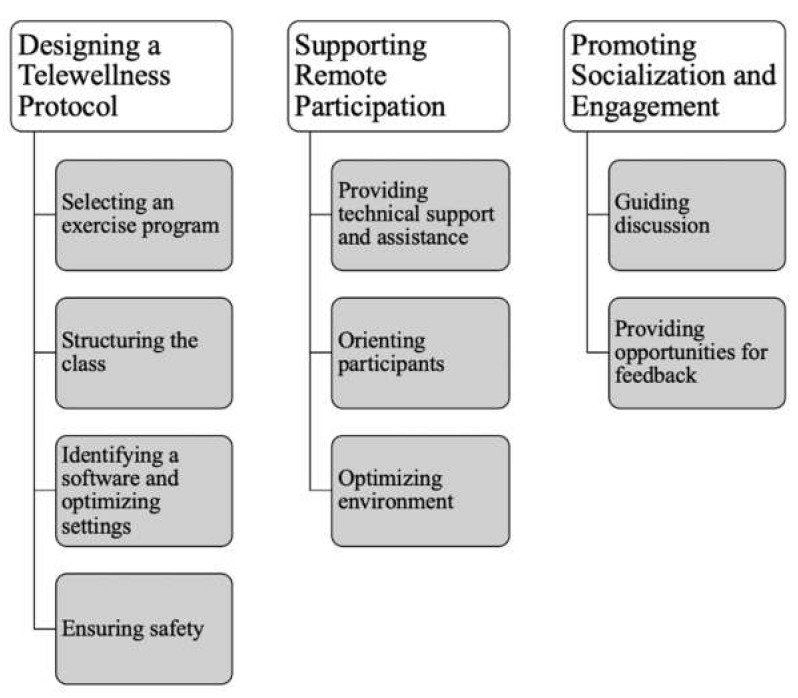
Research-Driven Guidelines for Delivering Group Telewellness Classes to Older Adults.

Through our iterative approach and the methods described above, we identified guidelines for delivering telewellness interventions to older adults, inclusive of those with long-term mobility disabilities (Figure 1). The guidelines focus on three aspects of the intervention: designing a telewellness protocol, supporting remote participation, and promoting socialization and engagement. The following results describe the guidelines in detail. These guidelines are also presented in the form of a step-by-step guide geared toward instructors and staff [47].

### 3.1. Designing a Telewellness Program

#### 3.1.1. Selecting an Exercise Program

In contrast to in-person exercise classes, participants in telewellness classes do not have an instructor who is physically there with them to provide any necessary adjustments, feedback, and support. As such, it is important to choose an exercise program that is safe and effective for the participant population, allows for modifications for a range of participant abilities, and is appropriate for remote delivery. Evidence-Based Programs (EBPs) are ideal choices for telewellness classes given they have been shown to be safe and effective. Videoconferencing holds great potential to expand access to existing EBPs.

There is great heterogeneity among older adults pertaining to physical and mobility capabilities. For some, standing for an extended period can be difficult or impossible. Therefore, for older adults—especially those with disabilities—seated exercise classes can provide a safer alternative to standing classes, as falls and fall-related injuries among older adults are more likely to occur with standing (vs. seated) exercises. Seated exercise classes can be a safe and effective method for individuals with upper and/or lower body mobility disabilities to engage in exercise. Older adults who participate in seated exercise classes can still receive many physical benefits [48]. Additionally, there is evidence to suggest that even visualizing physical activity in the absence of movement has some benefits [49] because the imagery of actions cause neural activations in similar areas of the brain as those used for executing actions [50]. Seated classes are also ideal for delivery via videoconferencing, as participants can place their device (i.e., computer, tablet, or smartphone) on a flat, stationary place in front of their chair (e.g., desk or table) and the single-camera angle view will capture their body from the waist up, which is often the primary focus of seated exercise.

Standing exercise classes can be delivered via videoconferencing, but there are important considerations to ensure the safety of participants. For standing classes, it is recommended that the instructor has a view of the participants’ full bodies to make sure they are moving safely. It may be difficult for participants to set up their device camera in such a way that they can be fully visible to the instructor. For example, participants may need assistance in identifying and setting the best camera angle for a yoga flow class that alternates from laying on a mat to standing upright. To achieve an appropriate setup, instructors can have a one-on-one meeting with participants via videoconferencing before conducting the first class. Adjustable device stands that tilt may also be helpful. To accommodate individuals with limited mobility, seated modifications of the standing exercise program should be offered. In addition, to accommodate those with visual limitations or disabilities, sufficient verbal cueing should be used to guide participants.

#### 3.1.2. Structuring the Class

In structuring a telewellness class, it is essential to consider the exercise instruction in terms of how it will be delivered (i.e., live or pre-recorded) and who is providing it. For telewellness classes with live instruction, the instructor would have the capability to demonstrate movements and interact with class participants in real-time. This live format offers flexibility for the flow and content of the exercise class. In contrast, for telewellness classes that use pre-recorded exercise lessons, participants practice along with an instructional video and do not receive real-time instruction or feedback. For this set-up, it is recommended that any non-instructor staff (e.g., facilitators) convening the class, review and familiarize themselves with the program in advance of the class so they are equipped to offer support to participants as needed.

Instructors should be well-versed in the program being offered so they can effectively teach, address questions, and provide modifications as needed. It can be helpful for the instructor to create a cheat sheet of cues to reference during classes. Instructors for EBPs or other established programs should be trained and certified. For telewellness classes, instructors are unable to provide in-person adjustments, so it is especially important that they provide verbal cues that guide participants through the exercise and help them adjust accordingly. During class, the class instructor or facilitator should observe the participants’ movements for any potential safety concerns, such as over-extending, that could cause pain or injury. For any movement that participants cannot do, or cannot do safely or without pain, the instructor can provide guidance on visualizing the movements given that will still provide some benefits without the risks of the physical movements [49].

Some videoconferencing platforms, like Zoom, allow the user to change their view settings so that videos of other participants can be enlarged or presented in different style panels. Alternatively, a computer device could be connected to a TV screen with an HDMI cable to increase the screen size for both the instructor and/or participants. If all participants cannot be seen on the screen simultaneously, the instructor should manually click to view the videos of all participants every few minutes. If the instructor notices a participant that needs to make an adjustment, the instructor should advise the full class on the adjustment, as opposed to correcting an individual, to avoid making a participant feel singled out.

#### 3.1.3. Identifying a Software and Optimizing Settings

**Figure 2:**
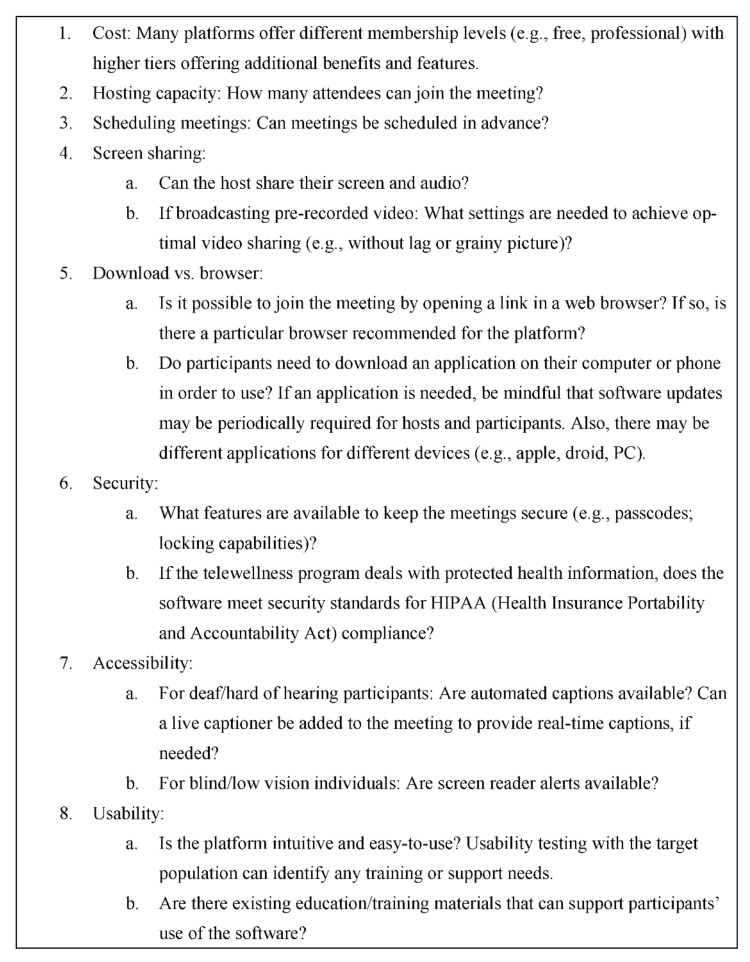
Considerations for Identifying a Software to Deliver Telewellness Classes.

It is necessary to identify the videoconferencing software that meets the specific needs and objectives of the class. There are a variety of videoconferencing platforms available, including some designed for general audiences (e.g., Zoom, Facetime, OneClick), as well as those specifically designed for healthcare purposes (e.g., Zoom for Telehealth; Doxy.me). Figure 2 highlights some key factors to consider in selecting a software platform for delivering Telewellness classes, including cost, specific features, and security.

The software settings will need to be determined to ensure the best quality user experience for the telewellness program, which depends on the delivery format (e.g., live streaming or pre-recorded) and the videoconferencing platform. Example settings to consider include video resolution, closed captioning, and audio input and output. For example, in the Tele Tai Chi study, we reduced the resolution of the video lessons being broadcasted to 540p, as we found this created an optimal balance between video quality and lag reduction. Closed captions were also enabled to support any participants with hearing limitations. In Zoom, we used the ‘share computer sound’ setting when beginning screen-sharing, as this directly shares the audio of our video lessons and avoids feedback from the microphone. Additionally, all participants were muted while the lesson was being broadcast.

#### 3.1.4. Ensuring Safety

There is a risk of injury with any type of physical activity. In conducting telewellness classes with older adults, especially those with disabilities, it is important to know if participants have health conditions or limitations that put them at a higher risk of injury from exercise. The Physical Activity Readiness Questionnaire (PAR-Q), developed by the National Academy of Sports Medicine, is one method commonly used to screen individuals before engaging in physical activity [51]. The self-report measure includes seven yes or no questions assessing whether individuals have certain high-risk factors, such as heart conditions, risk of stroke, or balance problems. If participants respond ‘Yes’ to any of the questions, they are advised to consult their doctor before participating in an exercise program.

Depending on participants’ health conditions, instructors may wish to require that they provide a letter or email from their healthcare provider approving their participation in the exercise class. Although this step can take time, the healthcare provider letter provides an additional level of confidence that participants can exercise safely. Additionally, it may be more efficient for smaller classes to bypass the PAR-Q and instead require all individuals to provide a healthcare provider letter before starting the class. The type of healthcare provider approval (e.g., physician, physician assistant, nurse practitioner, physical therapist) that is needed should be determined based on the specific program, the population, and the potential for risk. To facilitate the approval process, instructors can provide participants with key information about the exercise program (e.g., email, brochure, handout) to share with their healthcare provider.

Before participating in a telewellness program, participants should be fully informed about what to expect, including the potential risk of injury or harm. To help protect the class provider from potential claims or damages, it is advised that participants review and sign a form, such as a consent form or liability waiver, that indicates they are aware and assume the potential risks involved by participating. Given that telewellness classes are remote, it is helpful if instructors provide flexible options for participants to provide consent, such as completing an online form, scanning a photo of a signed form, or recording verbal consent. Instructors should also ask participants to provide an emergency contact whom the instructor can reach in the event of a fall, injury, or another type of concerning episode.

### 3.2. Supporting Remote Participation

#### 3.2.1. Providing Technical Support and Assistance

Older adult telewellness participants will likely be using different devices and have different levels of familiarity and proficiency with videoconferencing; for some, this could mean very minimal or even no prior experience. Providing technical support materials to participants in advance of class can help mitigate potential technology issues. A document should be provided with step-by-step instructions for how to join the class using the software, including screenshots of the tasks as a visual aid. It may also be helpful to create, or point to, troubleshooting Frequently Asked Questions (FAQs) to assist participants in navigating common issues (e.g., connecting speakers and microphone). Resources, such as these, should be easily accessible to participants to reference throughout the program. Hosting materials on a website can be a convenient way to share and update resources. Some participants may prefer to have hard copies of these resources sent to them via mail, so it is helpful to ask what works best for them.

During the actual class, the class instructor should do a quick ‘tech check’ to make sure each participant can see and hear them. Instructors should set aside time at the beginning of classes for troubleshooting, as some participants may still be learning how to use the software and experience technical difficulties; this troubleshooting time is especially useful early in the program. As participants become more comfortable joining the class, this time at the beginning of class can be a good opportunity to review some basic skills, such as using the mute/unmute or hide/unhide video features, or to teach some new technology skills (e.g., changing video backgrounds, using reactions, changing their view).

Depending on the class size, it may be difficult for the class instructor to simultaneously manage the session and any technical issues that can arise. An additional support staff member can be used as a tech moderator, whose role is specifically to handle the technical side of a class. This alleviates some of the tasks and stress put upon the class instructor, so they are more able to focus on leading the class. Some responsibilities might include: attending to technical issues that occur during the session; streaming the pre-recorded video lesson to the class; keeping track of participant attendance; taking notes. If the videoconference platform includes a chat box feature, it may be helpful to teach participants how to privately message the tech moderator if a technical issue or question arises to keep the conversation from disrupting the flow of the class and potentially distracting other participants.

#### 3.2.2. Orienting Participants

Conducting a 1-on-1 virtual orientation before the program can help ensure participants have the necessary technology setup to connect to the videoconferencing platform (e.g., Wi-Fi bandwidth, audio input/output settings, software downloads) and provide them with the opportunity to practice using it. Instructors can use this time to provide training on the software, for example, explaining all the actionable buttons on the interface that they will be using during class (i.e., mute/unmute) and having the participant practice them. An orientation session can also be a good time for instructors to discuss safety precautions, assess the participant’s needs for accommodations (e.g., closed captioning), and answer any remaining questions the participant may have about the program or how to join the class.

#### 3.2.3. Optimizing Environment

**Figure 3:**
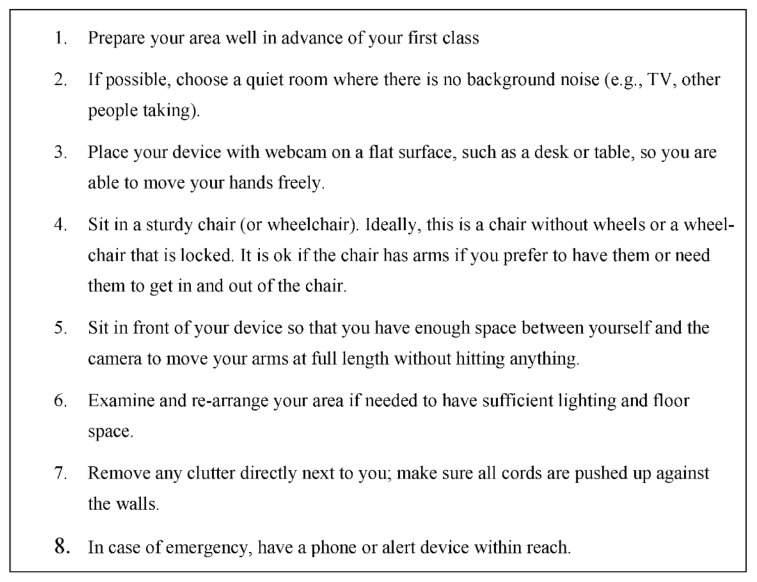
Environmental Setup Recommendations for Participants in Seated Telewellness Exercise Classes.

To help ensure safety and enhance the videoconferencing experience, participants should designate a space in their home to participate in the class. Figure 3 provides recommendations that participants can use to set up their home environment prior to engaging in a seated telewellness exercise class (e.g., location, device placement, chair type, lighting). In some cases, such as working with a more vulnerable population, instructors should consider conducting an environmental assessment via videoconferencing prior to the first class; this environmental assessment can be a component of an orientation session for the program, as described in the previous section. During this session, the instructor can observe the individual’s space, address any potential safety concerns, and help them optimize their audio/video quality so they can be seen and heard as clearly as possible.

### 3.3. Promoting Socialization and Engagement

In addition to physical benefits, telewellness classes can provide an opportunity to engage and socialize with other participants, allowing individuals to feel socially connected despite not being co-located. The opportunity for social interaction is especially important given the detrimental effects of social isolation. The extent to which participants can interact with the instructor and other class participants depends on group size and the goals of the program. For large group telewellness classes, it may not be feasible or effective for participants to have the capability to talk aloud to the instructor or attendees. Other methods for engaging participants in large group classes might include more passive methods, such as a list of who’s in class, a chat box, or the ability to recognize other participants with emoji symbols (e.g., clapping hands, heart).

#### 3.3.1. Guiding Discussion

Building in a discussion period can help establish rapport and facilitate relationships among participants. For programs that have time built-in for social interaction, a facilitator may be helpful to guide the discussion and make participants feel more at ease. This can be the class instructor or a support staff member. To help ensure the class discussion is respectful and enjoyable for all, it can be helpful to provide some guidelines for the conversation in advance. Guidelines should provide the ground rules for discussion, which might include: taking turns speaking; muting if there is background noise; passing if they do not want to participate or answer a specific question; avoiding language and topics that are potentially controversial or inappropriate for the class (e.g., politics, religion, medical advice). In the Tele Tai Chi study, we provide guidelines in the form of a Class Etiquette Guide, which is sent to participants at the beginning of the study.

The extent and timing of social discussion in a telewellness class will vary based on the goals of the specific class. Offering some time for participants to socialize before and after the exercise lesson mimics the flow of natural conversation that might occur at an in-person group class at a gym or senior center. This format also allows participants to greet one another and check-in before the exercise and debrief afterward. To guide the conversation, instructors can prepare weekly discussion topics and questions ahead of class. These questions should be designed to provide an opportunity to help participants get to know each other and to discuss topics relevant to the class.

During each Tele Tai Chi class, there is 10 min of social time before and after each exercise lesson. We developed a list of social time discussion topics for instructors to use. Pre-class topics focus on participants’ background (e.g., where they are from, favorite hobbies) as well as physical activity (e.g., challenges, personal goals, motivation). Post-class topics focus on the tai chi lesson (e.g., how the lessons are going/feeling, asking if there are questions about movements or modifications). These questions are intentionally less structured and formal so that participants can drive the discussion based on their needs. Based on our experience with the study, the format of 10 min of social time before and after the lesson works well for small group classes (max of eight people). Larger classes might consider offering less in-depth social discussion or using videoconferencing platforms with ‘breakout’ room capabilities to ensure all participants have time to respond to discussion questions and ask questions about the exercise.

The social interaction aspect of the Tele Tai Chi study has been successfully implemented as a part of the telewellness class. Given the benefits of increasing social opportunities for older adults and the benefits of including a social aspect in physical activity classes, telewellness interventions should strive to include a social aspect if possible.

#### 3.3.2. Providing Opportunities for Feedback

With in-person exercise classes, participants often have the opportunity to chat with instructors before and after class to provide feedback (e.g., likes, dislikes, movements that were particularly challenging). With telewellness classes, the opportunity to share questions, comments, or concerns can be less accessible unless it is intentionally built into the program. An online, post-class survey that is brief and easy to complete is an ideal way to elicit participant feedback in an automated way. There are a variety of survey platforms that can be used to create a post-class survey, including free options such as Google forms, as well as paid programs, such as Survey Gizmo and Qualtrics. Satisfaction with the class, instruction quality, and concerns are just a few potential topics that could be helpful to assess from class participants. At the end of each Tele Tai Chi class, tech moderators send the post-class survey link in the Zoom chat box and encourage participants to complete it. The post-class survey asks participants about: any technical issues they had (open-ended), how much they enjoyed the class (5 point scale from ‘not at all’ to ‘very much’), if they are comfortable moving on to the next lesson (Yes/No), the extent to which they feel ‘calm’ and ‘energized’ (10 point rating scale from ‘not at all’ to ‘very’), and how many times they have practiced tai chi on their own since the last class.

## 4. Discussion

Telewellness applications have much value for older adults who need accessible opportunities for physical activity and social engagement. In the U.S., the older adult population will likely continue to be the largest consumer group of healthcare given that both the number and proportion of older adults are rapidly growing [52]. Telewellness classes focused on physical activity have boomed recently, spawned by COVID-19 restrictions on in-person group gatherings. For example, April 2020 saw the biggest spike in downloads of Health and Fitness apps globally at 276 million, up 80% year over year and the evidence suggests this trend is continuing [53]. Unfortunately, most remote exercise programs are not designed for older adults or for those with diverse abilities and limitations, and most programs are not evidence-based. Additionally, most older adults (77%) report that they would need someone to help walk them through the process of using new technology [36]. Therefore, it is critical that telewellness interventions be designed with older adults’ needs and abilities in mind.

Just as telemedicine has increased access to clinical services, telewellness has the potential to increase access to wellness services, such as interventions for physical activity and social connectedness. Whereas telemedicine services are typically provided by a clinician, telewellness interventions may be delivered by a much broader array of professionals, including, but not limited to, exercise instructors, program staff at senior centers or senior housing residences, and clinicians. Worldwide, exercise instructors have been pivoting to deliver classes remotely with limited to no guidance on best practices for technical and environmental set-up. Therefore, the provision of support for instructors is just as important as for participants, given that they may have varying levels of experience.

Evidence-based interventions provide an ideal starting point for translation to a telewellness platform. Not only have they been shown to be efficacious, but they also can be paid for using Older Americans Act Title III-D Funding. One evidence-based intervention that has been tested with older adults with a range of abilities is Tai Chi for Arthritis [54,55,56]. It is well-suited as a telehealth intervention as it has a seated version that reduces the risk of falling, as well as being evidence-based for reducing falls, pain, and improving arthritis symptoms. To translate the in-person Tai Chi for Arthritis program into a telewellness intervention, we followed a human factors approach and user-centered design principles to facilitate ease of use and long-term adoption for target users (i.e., older adults with long-term mobility disabilities). As a result, we developed the research-driven telewellness class design guidelines presented here. These guidelines can be used by others to deliver additional telewellness classes to older adults with a wide range of abilities, including those with mobility disabilities. The guidelines include recommendations for the provision of support for older adults as well as instructors.

Interventions aimed at enhancing social connectedness in the aging population are widely advocated as a solution to the increasing rates of loneliness among older adults [57,58]. Most of these interventions provide education, such as social skill practice or social skill development, and offer the opportunity for group discussion [59,60,61,62,63]. Nevertheless, there is little documentation in the literature about telewellness applications aimed at social health. Our findings demonstrated the feasibility of adding a social component to a telewellness intervention and our guidelines provide researched-based recommendations on how to effectively do so.

Lastly, financial barriers may prevent some older adults from participating in telewellness programs. Just as wellness activities, such as in-person exercise classes, gym memberships, and massage, are reimbursable under some insurance policies (or are allowed expenses under some health savings accounts), insurance coverage should extend to telewellness classes and interventions given the associated positive health outcomes. Clinical effectiveness research on telewellness interventions will be instrumental in providing impetus for such coverage.

## 5. Conclusions

Given the importance of wellness activities on health outcomes, it is critical to ensure that they are accessible to older adults. Yet, many barriers prevent older adults from participating in in-person wellness activities and receiving the correlated health benefits. Translating in-person wellness activities, such as exercise, to telehealth platforms can overcome many of the barriers of in-person programs. This paper presented research-driven guidelines on delivering seated, group telewellness programs to facilitate the social and physical wellness of older adults with a wide range of abilities, including those with mobility disabilities. These guidelines can be used to expand access to socially-engaging, physical activity programs to older adults at home via videoconferencing, which can ultimately help support their physical health, mental health, and quality of life.

## Data Availability

Not applicable.
